# Mindful paths to food waste reduction: exploring the associations between gratitude, mindful eating behavior, and motivations for food waste avoidance

**DOI:** 10.3389/fpsyg.2025.1494653

**Published:** 2026-01-08

**Authors:** Kyriaki Giannou, Michail Mantzios

**Affiliations:** 1 Department of Psychology, Faculty of Health and Life Sciences, De Montfort University, Leicester, United Kingdom; 2 Department of Psychology, School of Health and Life Sciences, Birmingham City University, Birmingham, United Kingdom

**Keywords:** food waste avoidance, gratitude, mindful eating, mindful eating behavior, moderation analysis

## Abstract

Past research has emphasised the potential of gratitude in reducing food waste by prompting behavioral changes. A critical question, however, remains: can individuals experience gratitude without being attentive to the food they consume, and what are the implications for food waste? The present study investigated how gratitude influences motivations for food waste avoidance, and examined the moderating role of mindful eating as an agent of attentiveness. One hundred and sixty-nine UK adults completed self-report measures on mindful eating behavior, gratitude and food avoidance motives, and the results showed positive correlations between gratitude and moral and financial motivations to avoid food waste, as well as between mindful eating behavior and all motivations to avoid food waste. A moderation analysis showed that overall mindful eating behavior did not significantly moderate the relationship between gratitude and motivations to avoid food waste. Findings highlight the important associations between psychological and behavioral resources (i.e., gratitude and mindful eating) and the formation of motivations related to sustainable food practices. Implications for future research are discussed.

## Introduction

Urgent action is required to discover innovative solutions for food waste avoidance. Food waste poses significant challenges across social and economic domains globally, as research estimates that between 30% and 50% of food is disposed of in landfills, releasing harmful carbon dioxide and methane gases into the atmosphere (FAO, 2011; Stuart, 2009; Papargyropoulou et al., 2014; Yvon-Durocher et al., 2014). Additionally, the economic impact of food waste was estimated globally with an annual value of 1 trillion US dollars; an impact that is felt in nations like the United Kingdom with a 6-million-ton loss per year (Patel et al., 2021). Existing research predominantly focuses on cognitive information and consumer behavior, but interpersonal emotions, such as gratitude, have recently become a method of empowering individuals with a potential resource to mitigate food waste.

Gratitude is described as a sentiment of appreciation elicited by an experience that brings benefit, yet is not directly attributable to oneself (Emmons and McCullough, 2004), and is closely aligned to a “barometer” of action, motivation and reinforcement of moral conduct (McCullough et al., 2001). There are several research examples showing that gratitude fosters a sense of responsibility for sustainability (Liang and Guo, 2021; Kates and DeSteno, 2021; Tam, 2022), collectively highlighting how gratitude shapes both individual and collective behavior change. Recent research also explored the importance of campaigns, social marketing and education efforts to reduce food waste (Pearson and Perera, 2018; Reynolds et al., 2019; Stöckli et al., 2018), but Septianto et al. (2020) remains the only research example that links food waste reduction and gratitude, while other research signals short-lived implications for behavior change in food waste reduction (Zamri et al., 2020). To provide insights into an interpersonal emotion for behavior change, rather than strategies for campaigns or social marketing interventions, the present research expands on existing limited research on gratitude.

Yet, prior to behavior change, motivating individuals to avoid food waste may be of greater significance, and has only been a recent development in the field of food waste as an important driver of behavior change. Ribbers et al. (2023), interested in how consumers can be motivated to reduce waste, developed a Motivation to Avoid Food Waste scale (MAFW), capturing four primary motivations: environmental, moral, financial, and social. Ribbers et al. (2024) in later research further emphasised the significance of moral motivations in reducing household food waste, while Canova et al. (2024) found attitudes, norms, and perceived control predict food waste avoidance intentions, which in turn, drive behavior change. Collectively, such studies provide a multifaceted understanding of food waste dynamics, offering critical insights for further research and clarity on gratitude and its potential impact.

Beyond gratitude, eating behavior itself may be a significant component of fostering more sustainable behavior with food. Individuals often eat automatically and without conscious thought (Cohen and Farley, 2008; Peters, 2009), which automatically suggests a lower likelihood of appreciating (or being grateful of) the food, and consequently, being less inclined to preserve it for later. This lack of attention and awareness may reduce the enjoyment of food and weaken the potential link between gratitude and food waste avoidance. This is where mindful eating becomes a relevant construct. Mindful eating is a method employed within the field of health psychology, aims to counteract the automatic and distracted approach to food consumption, and is a “how to” method of bringing enjoyment into food consumption (Hong et al., 2011). Early research centered on mindful eating programs exploring both behaviors and decision making (e.g., Kristeller et al., 2014), while various recent studies focused specifically on *mindful eating behavior* (Mantzios, 2021; Mantzios, 2023a, 2023b), which specifically targets behavioral attentiveness and awareness during eating highlighted as being the core elements of mindful eating (e.g., Mantzios, 2021). Mindful eating behavior is defined as ‘the sustained attention to a sensory element of the eating experience (e.g., the taste) and a non-judgemental (or non-evaluative) awareness of thoughts and feelings that are incongruent to the sensory elements of the present eating experience’ (Mantzios, 2021, p. 369). Mantzios (2023a, 2023b) developed and validated a scale with two subscales (i.e., sensory attention and non-judgmental awareness) for measuring mindful eating behavior that is descriptive of the theory put forward in earlier literature on mindful eating behavior (Mantzios, 2021; Mantzios, 2025), and is in accordance to models of mindfulness and suggestions around the self-regulation of attention (Bishop et al., 2004; Kabat-Zinn, 2003). Significantly, enhanced mindful eating are suggested to promote feelings of appreciation and joy (Bays, 2017; Kawasaki and Akamatsu, 2020), which may serve as precursors to the experience of gratitude (Manela, 2016; Watkins et al., 2018), and consequently, influence motivations to reduce food waste. Investigating the behavioral trait of mindful eating could inform future research on how gratitude relates to food waste avoidance.

Therefore, the present explored the relationship between gratitude, motivations to reduce food waste, and mindful eating behavior, with mindful eating behavior specifically investigated as a potential moderator of the link between gratitude and these motivations.

## Methods

### Participants

One-hundred and sixty-nine participants were recruited through an online study platform. Participants’ age ranged from 21 to 68 years (*M* = 40.73, *SD* = 10.93), with the sample comprising 104 females and 65 males. One-hundred and fifty-seven participants identified as *White*, one as Black, three as *Asian*, six as *Mixed*, and two as “*Other*” without providing further information. Finally, participants’ self-reported height and weight assisted in the calculations of Body Mass Index (BMI) that ranged from 18.75 to 55.84 (*M* = 27.05, *SD* = 5.26). A power analysis using G*Power 3.1 determined that the required sample size for detecting a medium effect (f^2^ = 0.15) in a Model 1 moderation analysis, using *α* = 0.01 and power = 0.90, was 122 participants. Considering potential attrition rates and incomplete data entries, we recruited a larger number of participants.

### Materials

#### Motivation to avoid food waste (MAFW)

The 21-item MAFW scale (Ribbers et al., 2023) measures four motivations to avoid food waste: *environmental* (e.g., “Food waste has huge economic consequences for society”), *moral* (e.g., “Wasting food is disrespectful to poor people in this country”), *financial* (e.g., “Wasting food is a waste of my money”) and *social* (e.g., “I avoid food waste because I do not want other people to think I’m greedy”) motivations. Responses are provided according to what extent individuals personally avoid wasting food because of these motivations, with responses ranging from 1 (“*not at all*”) to 7 (“*very much*”). Ribbers et al. reported good internal reliability for all four motivations; *environmental, α* = 0.91; *moral*, *α* = 0.81; *financial*, *α* = 0.84; *social*, *α* = 0.92. In the present study, Cronbach’s alpha was also good for the four motivations: *environmental*, *α* = 0.93; *moral*, *α* = 0.83; *financial*, *α* = 0.87; *social*, *α* = 0.94.

#### The mindful eating behavior scale-trait (MEBS-T)

The 8-item MEBS-T scale (Mantzios, 2023b) measures two components of mindful eating behavior: *sensory attention* (e.g., “I fully taste what I am eating”) and *non-judgmental awareness* (e.g., “I hold my attention on what I am eating, despite recognising the occurrence of thoughts and/or feelings while I am eating”). Responses are provided on a 4-point Likert scale ranging from 1 (“*strongly disagree*”) to 4 (“*strongly agree*”). Mantzios reported good internal reliability for MEBS-T, for total score (*α* = 0.89) and subscales (*sensory attention*, *α* = 0.87; *non-judgmental awareness*, *α* = 0.86). In the present study, Cronbach’s alpha was equally good, for total score (*α* = 0.84) and subscales (*sensory attention*, *α* = 0.86; *non-judgmental awareness*, *α* = 0.83).

#### The gratitude questionnaire - six item form (GQ-6)

The GQ-6 (McCullough et al., 2002) measures a general grateful disposition (e.g., “if I had to list everything that I felt grateful for, it would be a very long list.”). Responses are provided on a 7-point Likert scale ranging from 1 (“*strongly disagree*”) to 7 (“*strongly agree*”). McCullough et al. reported good internal reliability for GQ-6, *α* = 0.82. In the present study, Cronbach’s alpha for GQ-6 was 0.82.

### Procedure

Participants were recruited through an online study advertisement platform (i.e., Prolific) and were compensated for their time (6.20 £/h). The study was conducted online through the Qualtrics experiment platform. Participants first responded to demographic questions about their age, sex, ethnicity, and provided their weight and height measurements (for BMI calculations). Participants then completed the self-report measures in a set order (i.e., WAFW, MEBS-T, and GQ-6).

### Data analysis

All statistical analyses were conducted using IBM SPSS 28. Bivariate correlations were examined to assess relationships between gratitude, mindful eating behavior, and motivations to avoid food waste. Moderation analyses were performed using PROCESS (Model 1) with 5,000 bootstrap samples, centering variables to their means. Simple effects coefficients were computed at three levels of the moderator (i.e., 1 SD below the mean, at the mean, and 1 SD above the mean). Statistical significance was determined at *p* ≤ 0.05, with bias-corrected confidence intervals (CI) used to confirm moderation effects. Partial *η*^2^ was used as an assessment of effect sizes, with benchmarks set at 0.01 (small), 0.06 (medium), and 0.14 (large) (Aiken et al., 1991; Hayes, 2017).

## Results

The present study examined relationships between gratitude, mindful eating behavior, and motivations to avoid food waste across different dimensions. Gratitude correlated positively with moral and financial motivations to avoid food waste, but not with social or environmental reasons. Mindful eating behavior positively correlated with overall gratitude and environmental, moral, financial, and social motivations to avoid food waste. Non-judgmental awareness showed similarly positive significant relationships between mindful eating behavior and all motivations to avoid food waste, while sensory attention showed significant positive relationships with environmental and moral motivations only (see Table 1).

**Table 1:** Bivariate correlations between mindful eating behavior, gratitude and motivations to avoid food waste.

Measures	MEBS-T	SA	NJA	Gratitude	ENV	MOR	FIN
MEBS-T	—						
SA	0.834^**^	—					
NJA	0.840^**^	0.401^**^	—				
GQ	0.208^**^	0.255^**^	0.094	—			
ENV	0.300^**^	0.233^**^	0.269^**^	0.134	—		
MOR	0.349^**^	0.315^**^	0.270^**^	0.313^**^	0.733^**^	—	
FIN	0.193^*^	0.148	0.175^*^	0.180^*^	0.311^**^	0.374^**^	—
SOC	0.250^**^	0.069	0.348^**^	0.025	0.436^**^	0.250^**^	0.173^*^

MEBS-T = Mindful eating behavior scale-trait; SA = mindful eating behavior scale-trait, sensory attention subscale; NJA = mindful eating behavior scale-trait, non-judgmental awareness subscale; motivation to avoid food waste subscales: ENV = environmental, MOR = moral, FIN = financial, and SOC = social; GQ = gratitude questionnaire. * <0.05 and ** <0.01.

Further moderation analyses explored the relationship between gratitude and motivation to avoid food waste, with mindful eating behavior as the moderator. Firstly, we focused on the overall mindful eating behavior scores and its moderation effects on the relationship between gratitude and four subscales of motivation to avoid food waste: environmental, moral, financial, and social motivations.

The moderation analyses revealed no significant interaction effects between mindful eating behavior and gratitude, and therefore, no significant moderation effects of mindful eating behavior on the relationships between gratitude and the four subscales of motivation to avoid food waste (environmental: *b* = 0.02, *p* = 0.610; moral: *b* = 0.02, *p* = 0.150; financial: *b* = 0.02, *p* = 0.240; social: *b* = −0.007, *p* = 0.840). Results indicated that the level of mindful eating behavior does not significantly alter the associations between gratitude and motivations to avoid food waste across these dimensions. For further exploratory moderation analyses with mindful eating subscales, please see Supplementary material S1.

## Discussion

The study aimed to explore the connections among gratitude, mindful eating behavior, and motivations to prevent food waste, investigating their interrelations and potential implications for waste reduction strategies. It was hypothesized that mindful eating behavior could moderate the relationship between gratitude and motivations to reduce food waste, including sub-dimensions of food waste reduction focusing on environmental, moral, financial, and social motivations.

Findings showed positive correlations between gratitude and both moral and financial motivations to avoid food waste. Such an association between gratitude and moral motivations was generally presumed (McCullough et al., 2001), but the association with financial motivations could be a consequence of an economic downturn and inflation in the UK during the recruitment period. Additionally, mindful eating behavior showed positive associations with gratitude and all motivations to prevent food waste. Findings from the mindful eating behavior subscales suggested that individuals with higher levels of non-judgmental awareness displayed a stronger relationship between gratitude and motivation to reduce food waste for moral and financial reasons than sensory attention. However, the study did not find significant moderation effects of overall mindful eating. While the moderation was not significant, the underlying constructs of mindful eating may be of further significance. A non-judgmental attitude is fundamental for self-regulating attention in mindfulness practices (Bishop et al., 2004; Kabat-Zinn, 2003), and may be instrumental in “the self-regulation of sensory attention while eating” (Mantzios, 2025), potentially diluted by investigating the overall score of mindful eating that includes the sensory attention subscale and was found to have weaker associations. Therefore, it is not simply a matter of paying attention to sensory elements of the food, but more about being able to regulate such attention. Apart from the quality of attention, another noteworthy implication is the potential health benefits when practising mindful eating (Mantzios et al., 2019, 2020) and the found experiences of improved eating behavior and regulated consumption, while gratitude-based interventions have mixed findings on either enhancing healthy eating behavior (Fritz et al., 2019), or a self-licencing or legitimising of consumption of energy-dense foods (see De Witt Huberts et al., 2012; De Witt Huberts et al., 2014; Schlosser, 2015). This is not to say that mindful eating literature does not suffer from similar problems (Mantzios, 2021), but a potential future direction could be the incorporation of a food gratitude scale (see Kawasaki et al., 2024), rather than a general gratitude trait scale. Overall, the present findings proposed that there are opportunities for collaborative interventions that merge food waste prevention with efforts to promote healthier eating (Cooper et al., 2018). Future research should explore the intersection of gratitude, via joy and appreciation of food, avoidance of food waste and mindful eating, which may explain explaining why mindful eating and gratitude are practical to mitigate food waste.

Several limitations should be acknowledged and considered before drawing solid conclusions. First, the self-reported nature of the research may have an overestimation or underestimation of motivations to avoid food waste. Future research should account for social desirability bias, as it may be a significant component associated with feelings of shame, which have been identified as an important determinant for food waste in previous research (Filimonau et al., 2025). Second, the cross-sectional design limits any causal interpretations and prevents directionality of relationships between gratitude, mindful eating, and food waste motivations. Third, while the sample size was adequate for statistical analyses, it may not be fully representative of broader populations, limiting generalisability. Fourth, the study did not account for potential external factors, such as economic status, education level, employment status, relationship status, or household food management practices, which may also impact food waste motivations and illustrate divergent or opposite findings. Future research should consider the limitations and explore experimental designs to further validate the present findings.

The present research shows the interaction between gratitude, mindful eating, and motivations to avoid food waste. While gratitude was positively associated with moral and financial motivations to reduce food waste, the moderation analysis did not show that mindful eating changed such relationships. Public awareness campaigns that integrate gratitude and/or mindful eating, or, public health programs targeting obesity, food insecurity, or malnutrition receiving food appreciation and mindful eating training are two potential policy-oriented examples of where such research could lead us. Despite the limitations, the present research provides valuable insights into how mindful eating and gratitude may contribute to sustainable food consumption practices, offering a promising avenue for both academic inquiry and real-world applications through mindful eating and gratitude practices.

## Data Availability

The raw data supporting the conclusions of this article will be made available by the authors, without undue reservation.
