# Supplementary material for: Mindful paths to food waste reduction: exploring the associations between gratitude, mindful eating behavior, and motivations for food waste avoidance
Source: Front Psychol. 2026 Jan 8;16:1494653. doi: 10.3389/fpsyg.2025.1494653 (PMC12823508; doi:10.3389/fpsyg.2025.1494653)
Supplement: Supplementary file 1 [file Supplementary_file_1.docx]

**Supplementary Exploratory Analyses on Mindful Eating Behaviour Subscales**

Follow-up moderation analyses were conducted using the subscales of mindful eating behavior: *sensory attention* and *non-judgmental awareness*. The *non-judgmental awareness* subscale moderated the relationships between gratitude and moral motivation (*b* = 0.08, *p* = .001, η^2^ = .014), and gratitude and financial motivation (*b* = 0.05, *p* = .030, η^2^ = .007) to avoid food waste. None of the other moderation analyses yielded significant results (Sensory Attention - environmental: *b* = -0.02, *p* = .790; moral: *b* = -0.01, *p* = .650; financial: *b* = -0.002, *p* = .930; social: *b* = 0.02, *p* = .700; Non-Judgmental Awareness - environmental: *b* = 0.09, *p* = .120; social: *b* = -0.01, *p* = .820).

Further exploration revealed that higher levels of *non-judgmental awareness* in mindful eating behavior strengthened the positive association between gratitude and moral and financial motivations to avoid food waste. This suggests that individuals with greater non-judgmental awareness may perceive and value their feelings of gratitude more strongly to motivate moral and financial aspects of avoiding food waste (see Table 2, Figures 1 and 2).

In summary, while the overall mindful eating behavior did not moderate the relationship between gratitude and motivations to avoid food waste, the specific facet of non-judgmental awareness within mindful eating behavior did exhibit significant moderation effects on the relationships between gratitude and moral, as well as financial motivations to avoid food waste. These findings shed light on the nuanced role of mindful eating behavior in shaping the connections between gratitude and motivations related to food waste avoidance.

*Table 2. Non-judgmental awareness moderating the relationship between gratitude and moral and financial reasons for avoiding food waste.*

|  | *Coefficient* | *SE* | *t* | *p* | *LLCI* | *ULCI* |
| --- | --- | --- | --- | --- | --- | --- |
| ***Moderation (DV: Moral)*** |  |  |  |  |  |  |
| *Gratitude* | *-.521* | *.233* | *-2.233* | *.027* | *-.981* | *-.060* |
| *MEB_NJA* | *-2.067* | *.795* | *-2.601* | *.010* | *-3.637* | *-.498* |
| *Gratitude × MEB_NJA* | *.077* | *.024* | *3.257* | *.001* | *.030* | *.123* |
| *-1SD* | *.078* | *.068* | *1.151* | *.252* | *-.056* | *.213* |
| *Average* | *.247* | *.053* | *4.635* | *<.001* | *.142* | *.352* |
| *+1SD* | *.416* | *.080* | *5.191* | *<.001* | *.258* | *.574* |
| ***Moderation (DV: Financial)*** |  |  |  |  |  |  |
| *Gratitude* | *-.364* | *.222* | *-1.639* | *.103* | *-.804* | *.075* |
| *MEB_NJA* | *-1.357* | *.758* | *-1.790* | *.075* | *-2.854* | *.140* |
| *Gratitude × MEB_NJA* | *.049* | *.022* | *2.194* | *.030* | *.005* | *.094* |
| *-1SD* | *.020* | *.065* | *.314* | *.754* | *-.108* | *.149* |
| *Average* | *.129* | *.051* | *2.533* | *.012* | *.028* | *.229* |
| *+1SD* | *.237* | *.076* | *3.104* | *.002* | *.086* | *.388* |

***
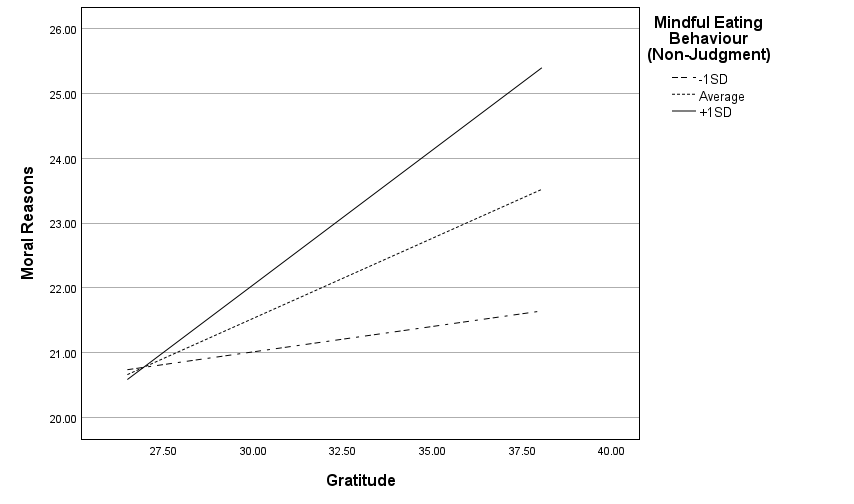
***

*Figure 1. Conditional effects of mindful eating behaviour (non-judgment subscale) on gratitude and moral reasons to avoid food waste.*

***
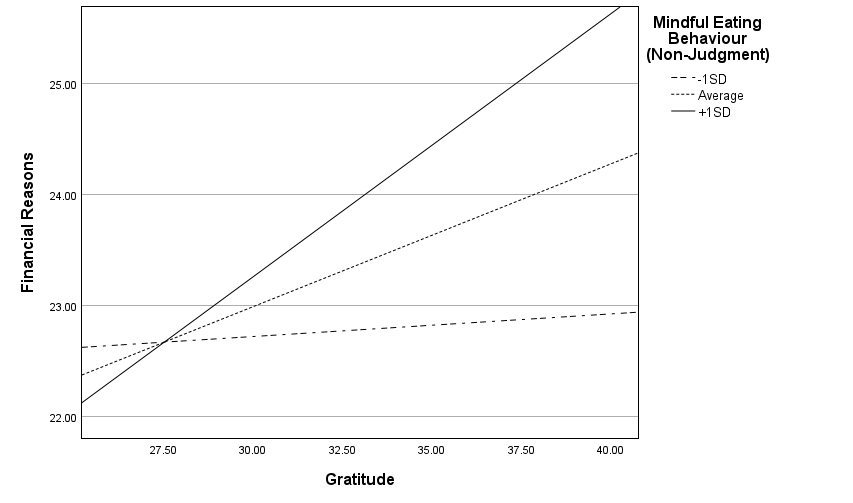
***

*Figure 2. Conditional effects of mindful eating behavior (non-judgment subscale) on gratitude and financial reasons to avoid food waste.*
